# 

**Published:** 1887-03

**Authors:** 


### THE BRISTOL

### Medico-Chirurgical Journal.

A JOURNAL OF THE MEDICAL SCIENCES FOR THE WEST OF ENGLAND AND SOUTH WALES

*PUBLISHED UNDER THE AUSPICES OF*

*THE BRISTOL MEDICO-CHIRURGICAL SOCIETY.*

EDITOR:

J. GREIG SMITH, M.A., F.R.S.E.

ASSISTANT-EDITOR:

L. M. GRIFFITHS.

---

"Scire est nescire, nisi id me

Scire alius sciret."

---

SURGEON GEN'L'S OFFICE LIBRARY 122067

*VOL. V.*

BRISTOL: J. W. ARROWSMITH;
LONDON: J. & A. CHURCHILL, 11 NEW BURLINGTON STREET.
1887.

WI
BR323

J.W. ARROWSMITH,

Printer and

Publisher,

QUAY STREET, BRISTOL

### CONTENTS OF VOL. V.

ORIGINAL PAPERS :— *Page.*

Ought Craniotomy to be Abolished ? By JOSEPH GRIFFITHS SWAYNE, M.D. .. ... ... ... ... ... ... ... ... ... 1

On Graves's Disease, with a Case. By J. MICHELL CLARKE, M.A., M.B. ... ... ... ... ... ... ... ... ... ... 17

Peripheral Neuritis. By J. MICHELL CLARKE, M.A., M.B. ... 73

On Rhinitis. By W. J. PENNY ... ... ... ... ... ... ... ... ... 95

Latent Rheumatism. By CHARLES MOORE JESSOP ... ... ... 153

Hoarseness and Loss of Voice. By BARCLAY J. BARON, M.B. ... 163

Shakspere and the Medical Sciences. By L. M. GRIFFITHS ... 225

Observations on Some of the New Hypnotics. By P. WM. MACDONALD, M.D. ... ... ... ... ... ... ... ... ... ... 257

CLINICAL RECORDS :—

Ill Effects of a Small Aneurism of the Aortic Arch Illustrated. By HENRY F. A. GOODRIDGE, M.D. ... ... ... ... ... 30

A Case of Suppurating Middle Ear, Thrombosis and Phlebitis of Lateral Sinus, Death. By W. H. HARSANT. ... ... ... 37

On a Case of Progressive Muscular Atrophy : Demonstrating the Nervous Origin and Nature of the Disease, and its Amenability to Nervine Tonics. By CHARLES WILLIAMS 103

Some Interesting Cases of Stone in the Bladder. By J. GREIG SMITH, M.A., F.R.S.E. ... ... ... ... ... ... ... ... 177

*Page.*

A Case of Tumour in the Orbit. By F. Richardson Cross, M.B. ... ... ... ... ... ... ... ... ... ... ... ... 189

On a Case of Cystic Goitre treated by "Shelling Out." By Charles Tanfield Vachell, M.D. ... ... ... ... ... ... 264

Case of Aneurism of Orbit. By A. W. Prichard ... ... ... 267

Treatment of Warts by Internal Administration of Arsenic. By Bingley G. Pullin ... ... ... ... ... ... ... ... ... ... 269

Periscope ... ... ... ... ... ... ... ... ... ... 43, 132, 211, 287

Notes on Preparations for the Sick ... ... ... 58, 124, 201, 282

Reviews of Books ... ... ... ... ... ... ... ... 64, 117, 194, 272

Scraps ... ... ... ... ... ... ... ... ... ... ... 71, 151, 223, 295

### Bristol Medico-Chirurgical Society.

---

### PAST PRESIDENTS.

1874—75. FREDERICK BRITTAN, M.D., B.A. Dub.

1875—76. ROBERT WILLIAM COE.

1876—77. SAMUEL MARTYN, M.D. Ed.

1877—78. AUGUSTIN PRICHARD, M.D. Berlin.

1878—79. HENRY EDWARD FRIPP, M.D. Würzburg.

1879—80. WILLIAM MICHELL CLARKE.

1880—81. JOSEPH GRIFFITHS SWAYNE, M.D. Lond.

1881—82. EDWARD LONG FOX, M.D. Oxon.

1882—83. JAMES GEORGE DAVEY, M.D. St. And.

1883—84. WILLIAM JOHNSTONE FYFFE, M.D. Dub.

1884—85. GEORGE FORSTER BURDER, M.D. Aberd.

1885—86. WILLIAM HENRY SPENCER, M.D., M.A. Cantab.

1887.

### LIST OF SUBSCRIBERS

TO THE

### Bristol Medico-Chirurgical Journal.

*Those marked * are Members of the Society.*

| *Name.* | *Residence.* |
|---|---|
| Ackland, J. Mackno | 24 Southernhay, Exeter. |
| Ackland, W. H., M.D. St. And. | Bideford. |
| Adams, George | 5 Oakfield Road, Clifton. |
| *Adams, John Barfield | 3 Selwood Villas, Bristol. |
| Alexander, James, M.D., M.Ch., B.A. Dub. | Bishop's Place, Paignton, Devonshire. |
| Alford, George Ernest | 5 Royal Crescent, Weston-super-Mare. |
| Alford, Henry J., M.D. Lond. | 2 Marlborough Terrace, Taunton. |
| Andrews, Onslow, M.D. St. And. | 159 White Ladies Road, Bristol. |
| Anstie, Thomas B. | 21 Northgate Street, Devizes. |
| *Atchley George F., M.B. Lond. | Tyndall's Park, Bristol. |
| Baker, A. de W. | 2 Lawn Terrace, Dawlish. |
| *Barclay, W. M. | General Hospital, Bristol. |
| Baretti, T. G. L'Enardi | 49 Royal York Crescent, Clifton. |
| Barnett, Lawrence, M.B. Lond. | 5 Walter Road, Swansea. |
| *Baron, Barclay J., M.B., C.M. Ed. | 12 Richmond Hill, Clifton. |
| Basset, Walter | The Infirmary, Newport, Mon. |
| Batten, Rayner W., M.D. Lond. | 1 Brunswick Square, Gloucester. |
| Batten, W. Smith | Curzon House, Calne. |
| Beale, Lionel S., M.B. Lond. | 61 Grosvenor Street, London, W. |
| Beatson, W. B., M.D. St. And. | 11 Cavendish Place, Bath. |
| *Beddoe, John, M.D. Ed., B.A. Lond., F.R.S. | The Manor House, Clifton. |
| Benham, Harry A., M.D., C.M. Aberd. | Asylum, Stapleton, Gloucestershire. |
| Bennett, W. S. | Escot, Penzance. |
| Berry, F. C., M.D., B.Ch., B.A. Dub. | Lyn Cottage, Lynton. |
| Bisdée, Alfred J. | Eversleigh, Banwell, Somersetshire. |
| *Blacker, A. E. | 5 Unity Street, Bristol. |
| Blacker, E. | Midsomer Norton. |
| *Blagg, Arthur F. | 6 West Mall, Clifton. |
| *Board, Edmund C. | 7 Caledonia Place, Clifton. |
| Bower, Ernest D. | 1 Norfolk Terrace, Gloucester. |
| Boyle, Thomas | New Quay, Cornwall. |
| Boys, Arthur Henry | Chequer Street, St. Alban's. |
| Bramwell, J. W., M.D. Ed. | 6 Bayshill Villas, Cheltenham. |
| Bright, J. A. | Glastonbury. |
| Broom, John, M.D. Brussels | St. Paul's Road, Clifton. |

### LIST OF SUBSCRIBERS.

| | |
|---|---|
| BROWN, G. ARTHUR ... ... ... ... ... ... ... | Tredegar. |
| *BROWN, WILLIAM, M.B., C.M. Glasg. ... ... | Fishponds, Gloucestershire. |
| BROWNE, A. E. NEWBURY ... ... ... ... ... | Burbage, Wiltshire. |
| BROWNE, G. HENRY ... ... ... ... ... ... ... | The Hermitage, Brynmawr, Breconshire. |
| BROWNE, J. WALTON, M.D., B.A. Qu. Univ. I. | 10 College Square North, Belfast. |
| BUBB, JOHN ... ... ... ... ... ... ... ... ... | 6 Royal Crescent, Cheltenham. |
| *BURDER, GEORGE F., M.D. Aberd. ... ... ... | 63 Oakfield Road, Clifton. |
| BURGES, R. E., M.D., M.Ch., B.A. Qu. Univ. I. | Kettering. |
| BURROUGHS, E. F. H. ... ... ... ... ... ... | Bristol Road, Weston-super-Mare. |
| *BUSH, J. PAUL ... ... ... ... ... ... ... ... | 2 Rodney Cottages, Clifton. |
| | |
| CANN, FRANCIS M. ... ... ... ... ... ... ... | Sefton House, Dawlish. |
| CAREY, J., M.D. Lond. ... ... ... ... ... ... | 2 Hamdon Villas, Taunton. |
| CARLYON, THOMAS B. ... ... ... ... ... ... | Redruth. |
| CARNALL, EDWARD ... ... ... ... ... ... ... | Fowey, Cornwall. |
| *CARR, ALEXANDER ... ... ... ... ... ... ... | Wells Road, Bristol. |
| CARTER, R. W., M.D. Calcutta ... ... ... ... | 1 Bank Buildings, Weymouth. |
| *CARTER, W. F.... ... ... ... ... ... ... ... | Coronation Road, Bristol. |
| CAVE, EDWARD J., M.D. Lond. ... ... ... ... | Royal United Hospital, Bath. |
| CLARK, THOMAS ... ... ... ... ... ... ... ... | Blair Lodge, Minehead, Somersetshire. |
| CLARKE, ARTHUR, B.A. Lond. ... ... ... ... | Street, Somersetshire. |
| CLARKE, FRED. H. ... ... ... ... ... ... ... | Holly Lodge, Chillington, Kingsbridge. |
| *CLARKE, JOHN MICHELL, M.B., M.A. Cantab. | 2 York Buildings, Clifton. |
| CLAY, CHALLONER ... ... ... ... ... ... ... | Manor House, Fovant. |
| CLAY, R. HOGARTH, M.D. Ed. ... ... ... ... | 4 Windsor Villas, Plymouth. |
| COATES, HARCOURT ... ... ... ... ... ... ... | 17 Endless Street, Salisbury. |
| COATHUPE, EDWIN W. ... ... ... ... ... ... | 7 Clifton Park, Clifton. |
| *COE, R. W. ... ... ... ... ... ... ... ... ... | 14 Berkeley Square, Bristol. |
| COKER, T. V. ... ... ... ... ... ... ... ... ... | 2 Chesterfield Place, Clifton. |
| COLE, THOMAS, M.D. Lond. ... ... ... ... | 18 Circus, Bath. |
| *COLLINS, H. W. ... ... ... ... ... ... ... ... | Wrington, Somersetshire. |
| COLLYNS, ROBERT J. ... ... ... ... ... ... | Dulverton, Somersetshire. |
| COLMAN, THOMAS J., M.D. Ed.... ... ... ... | Elmgrove Road, Bristol. |
| COLMER, PTOLEMY S. H., M.D. Dur. ... ... | South Street House, Yeovil. |
| COLTHURST, J. B. ... ... ... ... ... ... ... | Wincanton. |
| COOKE, A. S. ... ... ... ... ... ... ... ... ... | Badbrook House, Stroud. |
| COOPER, HERBERT ... ... ... ... ... ... ... | Hampstead, London, N.W. |
| *CORBOULD, GEORGE G. ... ... ... ... ... ... | 22 Berkeley Square, Bristol. |
| CORNWALL, JAMES ... ... ... ... ... ... ... | Fairford, Gloucestershire. |
| COWAN, F. S. ... ... ... ... ... ... ... ... ... | 27 Queen Square, Bath. |
| COX, WILLIAM I. ... ... ... ... ... ... ... ... | Hawkesbury-Upton. |
| CRESPI, ALFRED J. H. ... ... ... ... ... ... | St. John's Hill, Wimborne. |
| CRIPPS, EDWARD CHARLES ... ... ... ... ... | Charlton House, Cirencester. |
| CRISP, JAMES HENRY ... ... ... ... ... ... | The Grange, Lacock. |
| *CRISP, NATHANIEL ... ... ... ... ... ... ... | Keynsham, Somersetshire. |
| *CROSS, F. RICHARDSON, M.B. Lond. ... ... | Chandos Villa, Clifton. |
| *CROSSMAN, EDWARD, M.D. Dur. ... ... ... | Hambrook, Gloucestershire. |
| CURRIE, ANDREW S., M.D. Ed.... ... ... ... | The Moorlands, Lydney. |
| | |
| *DACRE, JOHN ... ... ... ... ... ... ... ... | Royal Infirmary, Bristol. |
| *DALY, GEORGE H., M.D. St. And. ... ... ... | Burley House, Chippenham. |
| DANIEL, T. P. ... ... ... ... ... ... ... ... | Beaminster, Dorsetshire. |
| *DAVEY, JAMES G., M.D. St. And. ... ... ... | Ellesmere House, Shepperton. |
| *DAVIES, D. S., M.B. Lond. ... ... ... ... | 60 Oakfield Road, Clifton. |
| DAVIES, E. CLUNEGLAS ... ... ... ... ... | Pontfaen, Lampeter, Cardiganshire. |

| | |
|---|---|
| DAVIES, JOHN W. ... ... ... ... ... ... ... | Ebbw Vale, Monmouthshire. |
| DAVIS, ROBERT ... ... ... ... ... ... ... ... | 14 St. James's Square, Bath. |
| DAVIS, THEODORE, M.D. Lond. ... ... ... | Beachcroft, Clevedon. |
| DAY, PERCY HOWARD ... ... ... ... ... ... ... | Stalmine Lodge, Poulton-le-Fylde. |
| DEAS, P. MAURY, M.B., M.S. Lond. ... ... | Wonford House, Exeter. |
| DENING, EDWIN ... ... ... ... ... ... ... ... | Stow-on-the-Wold. |
| DEVONALD, ALFRED ... ... ... ... ... ... ... | 13 Windsor Terrace, Penarth. |
| *DEW, HENRY R. ... ... ... ... ... ... ... ... | 25 Berkeley Square, Bristol. |
| *DOBSON, NELSON C. ... ... ... ... ... ... ... | 27 Victoria Square, Clifton. |
| *DOWSON, C. H. ... ... ... ... ... ... ... ... | Tyndall's Park, Bristol. |
| *DOWSON, W., M.B., M.A. Cantab. ... ... ... | Great George Street, Bristol. |
| DOYNE, ROBERT W. ... ... ... ... ... ... ... | Southbourne, Oxford. |
| DUNBAR, ELIZA L. WALKER, M.D. Zürich ... | 9 Oakfield Road, Clifton. |
| DUNCAN, WILLIAM ... ... ... ... ... ... ... ... | Ridgeway, Frome. |
| | |
| EADON, W. F. BAILEY ... ... ... ... ... ... ... | Hambrook, Gloucestershire. |
| *EAGER, REGINALD, M.D. Lond. ... ... ... | Northwoods, Winterbourne. |
| EAST, EDWARD ... ... ... ... ... ... ... ... | 16 Upper Berkeley Street, London, W. |
| EDDOWES, CHARLES ... ... ... ... ... ... ... | Maddington, Devizes. |
| EDGEWORTH, T. F.... ... ... ... ... ... ... ... | 1 Stokes Croft, Bristol. |
| EDWARDES, THOMAS ... ... ... ... ... ... ... | Llansaintffraid, Montgomeryshire. |
| *ELLIOTT, CHRISTOPHER, M.D., B.A. Dub. ... | 88 Pembroke Road, Clifton. |
| ELLIS, HENRY V., M.B., C.M. Aberd. ... ... | Ty Bryn, Reynoldstone. |
| *ELWIN, J. FOUNTAIN ... ... ... ... ... ... ... | 17 Clyde Road, Bristol. |
| EMBLETON, DENNIS C. ... ... ... ... ... ... ... | St. Wilfrid's, Bournemouth. |
| *ETCHES, W. R. ... ... ... ... ... ... ... ... | 13 Dowry Square, Clifton. |
| EVANS, G. M. ... ... ... ... ... ... ... ... ... | Bridport. |
| EVANS, J. FENTON, M.B., C.M. Ed. ... ... | 6 Whitehall Yard, London, S.W. |
| EVANS, W. G. ... ... ... ... ... ... ... ... ... | Beckington. |
| *EWENS, JOHN ... ... ... ... ... ... ... ... ... | 14 White Ladies Road, Clifton. |
| | |
| FAIRBANKS, W., M.D., C.M. Ed. ... ... ... | Wells, Somersetshire. |
| *FAWCETT, W. J., M.B., M.Ch., B.A. Dub. ... | Shirehampton. |
| *FENDICK, R. GEORGE ... ... ... ... ... ... ... | 3 Claremont Place, Clifton. |
| FERNIE, JAMES ... ... ... ... ... ... ... ... ... | Stratton St. Margaret. |
| FINCH, THOMAS, M.D. St. And. ... ... ... | Westville, St. Mary Church. |
| FISHER, FRED. B. ... ... ... ... ... ... ... ... | West Walk, Dorchester. |
| FLETCHER, HENRY STUDD ... ... ... ... ... | Lydbrook. |
| FORD, JOSEPH ... ... ... ... ... ... ... ... ... | Wedmore. |
| FORTY, D. H. ... ... ... ... ... ... ... ... ... | Wotton-under-Edge. |
| FOTHERGILL, J. MILNER, M.D. Ed. ... ... | 3 Henrietta Street, London, W. |
| FOX, ARTHUR E. W., M.B., C.M. Ed. ... ... | 16 Gay Street, Bath. |
| *FOX, BONVILLE B., M.D., M.A. Oxon. ... ... | Brislington. |
| FOX, CHARLES HENRY, M.D. St. And. ... ... | The Beeches, Brislington. |
| *FOX, E. LONG, M.D. Oxon. ... ... ... ... | Church House, Clifton. |
| FRASER, GRÆME B. ... ... ... ... ... ... ... | Belvidere, Weston-super-Mare. |
| FREEMAN, HENRY W. ... ... ... ... ... ... ... | 24 Circus, Bath. |
| FRY, JOHN B. ... ... ... ... ... ... ... ... ... | Swindon, Wiltshire. |
| FULLER, J. ... ... ... ... ... ... ... ... ... ... | Long Ashton. |
| FURSE, EDWIN ... ... ... ... ... ... ... ... ... | South Molton. |
| *FYFFE, W. JOHNSTONE, M.D., B.A. Dub. ... | 2 Rodney Place, Clifton. |
| | |
| GABB, J. EDWARD ... ... ... ... ... ... ... ... | London Road, Cheltenham. |
| GAMBLE, C. H.... ... ... ... ... ... ... ... ... | Barnstaple. |
| GARDINER, GEORGE ... ... ... ... ... ... ... | 1 Redcliff Hill, Bristol. |

### LIST OF SUBSCRIBERS.

| | |
|---|---|
| *Gibbs, Alfred N. Godby | 27 Chandos Road, Bristol. |
| Gibbs, Robert | Orchard House, Teignmouth. |
| Gilbertson, Richard | Aberystwith. |
| *Gloag, G. A. | 6 College Green, Bristol. |
| Goodden, Wyndham C. | 86 Stapleton Road, Bristol. |
| Gooding, J. Callender, M.D. Ed. | Berkeley Street, Cheltenham. |
| Goodridge, Henry F. A., M.D. Lond. | 10 Brock Street, Bath. |
| Goss, T. Biddulph | 1 Circus, Bath. |
| Gould, A. Pearce, M.B., M.S. Lond. | 16 Queen Anne Street, London, W. |
| Gowing, B. C. | Weirfield, Penistone. |
| Grace, Alfred | Chipping Sodbury. |
| Grace, Edward Mills | Thornbury, Gloucestershire. |
| Grace, Henry | Kingswood, Bristol. |
| Grace, W. G. | 57 Stapleton Road, Bristol. |
| Gray, F. A. | Ottery St. Mary. |
| Green, F. K. | 3 Gay Street, Bath. |
| Gregory, George S. | Southampton Row, London. |
| *Griffiths, L. M. | 9 Gordon Road, Clifton. |
| Grigor, Macadam | Alexandra Avenue, London, S.W. |
| Grote, G. W., M.B. Toronto | 11 Clare Gardens, Cardiff. |
| Guinness, T. A. | Bampton, Devonshire. |
| | |
| *Hailes, Clements D. G., M.D., C.M. Ed. | 11 King's Parade, Clifton. |
| Hale, Edmund T. | The Grange, Chew Magna. |
| Harper, Joseph | Bear Street, Barnstaple. |
| Harries, Evan | Rhiwlwyd, Pencader, Carmarthenshire. |
| *Harrison, A. J., M.B. Lond. | Guthrie Road, Clifton. |
| *Harsant, W. H. | 16 Pembroke Road, Clifton. |
| Hawkins, Cæsar F. | 7 Berkeley Square, Bristol. |
| Haydon, Edgar, M.B., C.M. Glasg. | The Laurels, Newton Abbot. |
| Hayman, A. G. | 1 Pembroke Road, Clifton. |
| Heane, W. C. | Cinderford. |
| Hearder, G. J., M.D. St. And. | Joint Counties Asylum, Carmarthen. |
| *Heaven, John C. | 52 Queen Square, Bristol. |
| *Herapath, C. K. C. | 11 Brunswick Square, Bristol. |
| *Hetling, H. E. | Stapleton Road, Bristol. |
| Hichens, James Stacey | 17 Green Lane, Redruth. |
| Hills, Frederic F. | 199 Cheltenham Road, Bristol. |
| Hinton, Joseph | Warminster. |
| Hoblyn, Francis Parker | 10 Bladud Buildings, Bath. |
| Hooker, Charles P. | 104 Dyer Street, Cirencester. |
| Hopkins, H. Culliford | 4 Gay Street, Bath. |
| Hospital, Bristol General (*Sec.*, W. Thwaites). | |
| Howells, William, M.B., C.M. Glasg. | Church House, Talgarth, Breconshire. |
| Howse, William | 8 London Street, Swindon, Wiltshire. |
| Howsin, E. Arthur, M.D. St. And. | Stroud. |
| Hudson, H. R. | 182 Commercial Road, Newport, Mon. |
| Hunt, A. D. | Millbrook House, Chagford. |
| Hyatt, Brownlow N. | Park View, Shepton Mallet. |
| | |
| *Imlay, G. A., M.B., C.M. Aberd. | 4 Apsley Villas, Bristol. |
| Infirmary, Bristol Royal (*Librarian*, Dr. James Swain). | |
| Institution, Liverpool Medical (*Hon. Treas.*, Dr. James Barr). | |
| *Isaac, G. Washington, M.B., C.M. Ed. | 8 King's Parade, Clifton. |

### LIST OF SUBSCRIBERS.

| | |
|---|---|
| JACKSON, J. B., M.D., M.Ch.Roy.Univ.I. ... | 56 Lemon Street, Truro. |
| JAMES, J. BOWEN ... ... ... ... ... ... ... | Woodlands, Aberaman, Aberdare. |
| JENKINS, JOHN HENRY ... ... ... ... ... ... | Liskeard. |
| JESSOP, CHARLES MOORE ... ... ... ... ... | 98 Sutherland Gardens, London, W. |
| JONES, E. R. ... ... ... ... ... ... ... ... | Cilgynlle-fawr, New Quay, Cardiganshire. |
| JONES, EVAN ... ... ... ... ... ... ... ... | Ty-mawr, Aberdare. |
| JONES, W. R., M.B., C.M. Glasg. ... ... ... | Senny Bridge, Breconshire. |
| JOYNES, FRANCIS JAMES ... ... ... ... ... | Eagle House, Dursley. |
| *KEALL, W. P. ... ... ... ... ... ... ... ... | Coronation Road, Bristol. |
| KEMPE, ARTHUR W. ... ... ... ... ... ... | 9 Southernhay, Exeter. |
| KENDALL, BERNARD ... ... ... ... ... ... | Elm Grove, Tenby. |
| KERR, ELIAS W., M.B., M.Ch., B.A. Dub. ... | Cerne Abbas. |
| KESTEVEN, W. B., M.D. St. And. ... ... ... | Little Park, Enfield. |
| KING, LOUIS J. ... ... ... ... ... ... ... ... | 10 Russel Street, Bath. |
| KINNEIR, R. ... ... ... ... ... ... ... ... | The Tower House, Malmesbury. |
| KIRKMAN, J. MILLER ... ... ... ... ... ... | Wootton Bassett. |
| LAING, W. A. GORDON, M.B., C.M. Glasg. ... | Boutport Street, Barnstaple. |
| LAMBE, J. ... ... ... ... ... ... ... ... ... | West Villa, Shurdington. |
| *LANSDOWN, F. POOLE ... ... ... ... ... ... | 19 White Ladies Road, Clifton. |
| *LAWRENCE, A. E. AUST, M.D., C.M. Aberd. . | 19 Richmond Hill, Clifton. |
| *LAWRENCE, HENRY ... ... ... ... ... ... | 9 Park Row, Bristol. |
| *LAXTON, HENRY ... ... ... ... ... ... ... | 12 Apsley Road, Clifton. |
| LEACH, J. COMYNS, M.D. Dur., B.Sc. Lond. . | Sturminster Newton. |
| LEAMON, MICHAEL T. ... ... ... ... ... ... | 1 Bedford Villas, Tavistock. |
| *LEES, E. LEONARD, M.B., C.M. Ed. ... ... | Children's Hospital, Bristol. |
| LEWELLYN, JOHN ... ... ... ... ... ... ... | Caerphilly. |
| LIBRARY, BRISTOL MUSEUM AND ... ... ... | Queen's Road, Bristol. |
| LIBRARY, CHELTENHAM ... ... ... ... ... | 5 Royal Crescent, Cheltenham. |
| LIDDON, WILLIAM, M.B. Lond. ... ... ... ... | Church Square, Taunton. |
| LILLEY, G. HERBERT, M.D. Brussels ... ... | H.M. Convict Prison, Portland. |
| LISTER, SIR JOSEPH, Bart., F.R.S. ... ... ... | 12 Park Crescent, London, N.W. |
| LLOYD, WALTER E. ... ... ... ... ... ... | 60 York Road, Bristol. |
| LODGE, JOHN ... ... ... ... ... ... ... ... | Keynsham, Somersetshire. |
| LOGAN, FREDERIC T. B. ... ... ... ... ... | Dean Lane, Bristol. |
| LOVELL, W. DAY ... ... ... ... ... ... ... | The Abbey, Bradford-on-Avon. |
| LOVELL, WILLIAM FREDERICK ... ... ... ... | Compton Martin, Somersetshire. |
| LOWE, T. PAGAN ... ... ... ... ... ... ... | 1 Alfred Street, Bath. |
| LUCKHAM, L. S. ... ... ... ... ... ... ... | General Infirmary, Salisbury. |
| *LUFFMAN, W. C. ... ... ... ... ... ... ... | 8 Cotham New Road, Bristol. |
| *LYONS, A. DE COURCY, M.B., C.M. Aberd. ... | Henley Lodge, Yatton, Somersetshire. |
| *LYSAGHT, W. C. ... ... ... ... ... ... ... | 13 Frederick Place, Clifton. |
| MACDONALD, P. W., M.D., C.M. Aberd. ... | County Asylum, Dorchester. |
| MACLEAN, J. CAMPBELL, M.B., C.M. Ed. ... | Swindon House, Swindon, Wiltshire. |
| MACLEAN, KENNETH ... ... ... ... ... ... | Greek Street, Stockport. |
| MANNING, G. H., M.B., B.Ch., B.A. Dub. ... | Combe Martin. |
| MANNING, H. J., B.A. Lond. ... ... ... ... | Laverstock House, Salisbury. |
| MARLEY, HENRY F. ... ... ... ... ... ... | The Nook, Padstow, Cornwall. |
| MARSH, CHARLES J. ... ... ... ... ... ... | Yeovil. |
| MARSH, O. E. BULWER ... ... ... ... ... | 35 Bridge Street, Newport, Mon. |
| *MARSHALL, HENRY, M.D. Ed., F.R.S.E. ... | 28 Caledonia Place, Clifton. |
| *MARTIN, E. F., M.B. Ed. ... ... ... ... ... | Victoria House, Weston-super-Mare. |

### LIST OF SUBSCRIBERS.

| | |
|---|---|
| Martin, Theodore | Temple Cloud. |
| Mason, Frederick | 20 Belmont, Bath. |
| Mason, S. B. | 12 Park Terrace, Pontypool. |
| Mason, William | Sedgemoor College, St. Austell. |
| *Matthews, Charles E. | 31 Pembroke Road, Clifton. |
| *Mayor, T. O. | 40 Apsley Road, Clifton. |
| McNicoll, John | High Street, Poole. |
| *Mead, H. Rimmington | Fishponds, Gloucestershire. |
| Meredith, John, M.D. Ed. | Wellington, Somersetshire. |
| Metford, J. Seymour | 31 Berkeley Square, Bristol. |
| Michell, George A. | Redruth. |
| Milne, John, M.B., C.M. Aberd. | 97 Clarence Road, Bristol. |
| Milward, James | 54 Charles Street, Cardiff. |
| Morgan, John | Langport, Somersetshire. |
| Morgan, William, M.D. Aberd. | 16 Edwards Terrace, Cardiff. |
| Morris, J. A. | Caerleon, Monmouthshire. |
| Moynan, W. Arthur, M.D., M.Ch. Qu. Univ. I. | St. Ann's Heath, Chertsey. |
| Mudge, T. | Towerhill, Bodmin. |
| Munden, Charles | Ilminster. |
| Murray, William, M.B., C.M. Ed. | Staple Hill, Gloucestershire. |
| | |
| Napier, T. W. A., M.B., C.M. Aberd. | Church Street, Egremont, Cheshire. |
| *Newman, Charles | 19 Portland Square, Bristol. |
| *Newnham, W. H. C., M.B., M.A. Cantab. | General Hospital, Bristol. |
| Newstead, James | 9 York Place, Clifton. |
| Newton, Charles John | Oriel Lodge, Cheltenham. |
| Nicholson, T. D., M.D., C.M. Ed. | 2 White Ladies Road, Clifton. |
| Norman, George | 12 Brock Street, Bath. |
| Norman, J. H. | Goodleigh, Winkleigh, Devonshire. |
| Norman, J. W. | Edde Cross House, Ross. |
| *Norton, J. A., M.D., C.M. Aberd. | 8 Redcliff Hill, Bristol. |
| *Norton, T. Chalmers | 12 King Square, Bristol. |
| Nourse, W. J. Chichele | Morchard Bishop, Devonshire. |
| | |
| Ogston, Alexander, M.D., C.M. Aberd. | 252 Union Street, Aberdeen. |
| Ollerhead, T. J. | Hillbury, Minehead, Somersetshire. |
| *Ormerod, Henry | Westbury-upon-Trym. |
| | |
| Padley, George | 2 Northampton Terrace, Swansea. |
| Paine, William Henry, M.D. Aberd. | Stroud. |
| Palm, Theobald A., M.D., C.M., M.A. Ed. | Greenhill, Thorncombe. |
| Palmer, F. Craddock | The Corderries, Chalford. |
| Parry, J. H. | 99 Ashley Road, Bristol. |
| Parsloe, H. H. | Black Torrington, Devonshire. |
| *Parson, T. Cooke | 21 White Ladies Road, Bristol. |
| Parsons, Francis J. C. | King Square, Bridgwater. |
| Parsons, Frederick | Common Hill, Frome. |
| Pauli, G. C. P. | 240 York Road, Bristol. |
| Paull, Josiah | Camborne, |
| Pearce, Edward | Camelford. |
| Pearse, Fred. E., M.D. St. And. | Argyle House, Frome. |
| *Penny, W. J. | 42 Caledonia Place, Clifton. |
| Pentland, Young J. | 11 Teviot Place, Edinburgh. |
| Perkins, J. H., M.D. New York | 14 Victoria Square, Clifton. |

### LIST OF SUBSCRIBERS.

| | |
|---|---|
| Peskett, A. W. C., M.B., B.C., M.A. Cantab. | Burnham, Somersetshire. |
| Phelps, W. Harford G., M.D. Aberd. ... | Beachfield, Weston-super-Mare. |
| Phillips, E. England ... ... ... ... ... ... | Broadwater House, Southend. |
| Phillips, J. W. ... ... ... ... ... ... ... ... | Cowbridge. |
| Philpots, Edward P., M.D., C.M. Aberd. ... | Bourne Hall, Bournemouth. |
| Philpots, John R. ... ... ... ... ... ... | Moorcroft, Parkstone, Dorsetshire. |
| *Pickering, C. F. ... ... ... ... ... ... ... | 12 Berkeley Square, Bristol. |
| Pippette, Walter... ... ... ... ... ... ... | Wilton, Wiltshire. |
| Pizey, G. F. P.... ... ... ... ... ... ... ... | Rydal Villa, Clevedon. |
| Plaister, W. H. ... ... ... ... ... ... ... ... | High Road, Tottenham. |
| *Pountney, W. E., M.D., C.M. Ed. ... ... | 107 White Ladies Road, Bristol. |
| Powell, William, M.B. Lond. ... ... ... | Hill Garden, Torquay. |
| Price, William, M.B. Lond. ... ... ... ... | 40 Park Place, Cardiff. |
| *Prichard, Arthur W.... ... ... ... ... ... | Gordon Road, Clifton. |
| *Prichard, Augustin, M.D. Berlin ... ... ... | 4 Chesterfield Place, Clifton. |
| *Prichard, J. E., M.B., B.A. Oxon. ... ... | 243 Cheltenham Road, Bristol. |
| Prideaux, T. Engledue P. ... ... ... ... | Wellington, Somersetshire. |
| *Pring, Arthur, B.A. Cantab. ... ... ... ... | 4 Pembroke Road, Clifton. |
| Prowse, Albert P. ... ... ... ... ... ... | Yennadon, Horrabridge, Devonshire. |
| *Prowse, Arthur B., M.D. Lond. ... ... ... | 4 Rodney Cottages, Clifton. |
| Pullin, Bingley G. ... ... ... ... ... ... | Sidmouth. |
| | |
| Randolph, Charles ... ... ... ... ... ... | St. Michael's, Milverton, Somersetshire. |
| Rawlings, John Adams ... ... ... ... ... | 4 Northampton Terrace, Swansea. |
| Reader, Jeremiah ... ... ... ... ... ... ... | Lyme Regis. |
| Redwood, T. Hall, M.D., C.M., M.A. Dur.... | The Lawn, Rhymney, Monmouthshire. |
| Rendall, John... ... ... ... ... ... ... ... | Forestside, Lymington. |
| Robathan, G. B. ... ... ... ... ... ... ... ... | The Grove, Risca. |
| Roberts, J. S. H. ... ... ... ... ... ... ... ... | 6 Dynevor Place, Swansea. |
| Rogers, George, M.D. St. And. ... ... ... | 17 Portland Square, Bristol. |
| Rolston, G. T. ... ... ... ... ... ... ... | 8 Osborne Villas, Stoke, Devonport. |
| Rolston, John R. ... ... ... ... ... ... ... ... | 5 St. Aubyn Street, Devonport. |
| Rossiter, George F., M.B. Lond. ... ... | Cairo Lodge, Weston-super-Mare. |
| *Roué, W. Barrett, M.D., M.S. Dur. ... ... | 2 Buckingham Place, Clifton. |
| Row, Charles ... ... ... ... ... ... ... ... ... | Lostwithiel. |
| Row, William ... ... ... ... ... ... ... ... ... | Lerrin, Lostwithiel. |
| Rowe, Edmund L.... ... ... ... ... ... ... ... | County Asylum, Gloucester. |
| *Roxburgh, Robert, M.B. Ed. ... ... ... ... | 1 Victoria Buildings, Weston-super-Mare. |
| *Rudge, C. King ... ... ... ... ... ... ... ... | 145 White Ladies Road, Bristol. |
| *Rudge, H. T. ... ... ... ... ... ... ... ... ... | 5 Colston Parade, Bristol. |
| Rundle, Edmund ... ... ... ... ... ... ... ... | Royal Cornwall Infirmary, Truro. |
| Rundle, Henry ... ... ... ... ... ... ... ... | 11 Clarence Parade, Southsea, Hampshire. |
| Runnalls, H. Boyle ... ... ... ... ... ... ... | Laurel Bank, Saltash, Cornwall. |
| Russell, M. W. H. ... ... ... ... ... ... ... | 6 Whitehall Yard, London, S.W. |
| | |
| Salmon, W. G. ... ... ... ... ... ... ... ... | Thornbury, Gloucestershire. |
| Scott, James, M.B., C.M. Ed.... ... ... ... | H.M. Prison, Hyde Road, Manchester. |
| Scott, Richard J. H. ... ... ... ... ... ... ... | 13 Bladud Buildings, Bath. |
| Scott, W. G., M.B., C.M. Ed. ... ... ... | Newton Abbot. |
| Searle, George C. ... ... ... ... ... ... ... | Brixham, Devonshire. |
| Searle, Richard Burford ... ... ... ... | St. Just. |
| Seward, W. J., M.B. Lond. ... ... ... ... | Asylum, Colney Hatch, London, N. |
| Sewart, J. H. ... ... ... ... ... ... ... ... ... | Lostwithiel. |
| Shapter, Lewis, M.D., B.A. Cantab. ... ... | The Barnfield, Exeter. |
| *Shaw, J. E., M.B., C.M. Ed. ... ... ... ... | 11 Lansdown Place, Clifton. |

| | |
|---|---|
| Sheen, A., M.D. St. And. ... ... ... ... ... | 23 Newport Road, Cardiff. |
| Sheldon, T. Steele, M.B. Lond. ... ... ... | Parkside House, Macclesfield. |
| *Sheppard, E. J. ... ... ... ... ... ... ... | 16 Park Place, Clifton. |
| *Shorland, Walter E. ... ... ... ... ... | 189 Cheltenham Road, Bristol. |
| Shortridge, Thomas Wood, M.D. Brussels | Honiton. |
| Sincock, J. Bain ... ... ... ... ... ... ... | Friarn Street, Bridgwater. |
| Skeate, Edwin ... ... ... ... ... ... ... | 16 Paragon, Bath. |
| *Skelton, Henry, M.D. St. And. ... ... ... | Downend, Gloucestershire. |
| *Skerritt, E. Markham, M.D., B.S., B.A. Lond. | Richmond Hill, Clifton. |
| *Skinner, Stephen, M.B., C.M. Aberd. ... | Ferndale, Clevedon. |
| *Smith, G. Munro ... ... ... ... ... ... ... | 70 Pembroke Road, Clifton. |
| *Smith, J. Greig, M.B., C.M., M.A. Ab., F.R.S.E. | 16 Victoria Square, Clifton. |
| Smith, J. Sidney, M.D. St. And. ... ... ... | Tiverton. |
| *Smith, R. Shingleton, M.D., B.Sc. Lond. | 9 Richmond Hill, Clifton. |
| Smith, Samuel ... ... ... ... ... ... ... | 7 Kingsdown Parade, Bristol. |
| Society, Cardiff Medical (*Hon. Sec.*, T. Garrett Horder). | |
| Society, Plymouth Medical (*Hon. Sec.*, C. Whipple). | |
| Society, Royal Medical and Chirurgical | 53 Berners Street, London, W. |
| Somer, James ... ... ... ... ... ... ... ... | Broadclyst. |
| *Spencer, W. H., M.D., M.A. Cantab. ... ... | 5 Lansdown Place, Clifton. |
| Spender, John K., M.D. Lond. ... ... ... | 17 Circus, Bath. |
| Stansfeld, G. M. ... ... ... ... ... ... ... | 19 Victoria Square, Clifton. |
| Statham, R. Whiteside ... ... ... ... ... | Cheddar, Somersetshire |
| Steel, Arthur R.... ... ... ... ... ... ... | Beer Alston, Devonshire. |
| Steel, S. H., M.B. Lond. ... ... ... ... ... | Abergavenny. |
| *Steele, Charles, M.D. Dur. ... ... ... ... | 17 Richmond Hill, Clifton. |
| Steele-Perkins, E. B. ... ... ... ... ... | 1 Hill's Buildings, St. Sidwell's, Exeter |
| *Stewart, James, B.A. Qu. Univ. I. ... ... | Dunmurry, Stoke Bishop. |
| Stockwell, Fred., M.D. Lond. ... ... ... | Bruton, Somersetshire. |
| Stoddart, F. Wallis ... ... ... ... ... ... | 1 Park Street, Bristol. |
| Storry, Frederick W. ... ... ... ... ... | Lansdown, Stroud. |
| *Swain, James, M.B. Lond. ... ... ... ... ... | Royal Infirmary, Bristol. |
| *Swayne, J. G., M.D. Lond. ... ... ... ... | 74 Pembroke Road, Clifton. |
| *Swayne, S. H.... ... ... ... ... ... ... ... | 129 Pembroke Road, Clifton. |
| Sydenham, George F. ... ... ... ... ... | Brushford, Exbridge. |
| | |
| Tait, Lawson ... ... ... ... ... ... ... ... | 7 The Crescent, Birmingham. |
| *Taylor, J. ... ... ... ... ... ... ... ... ... | 5 Ashley Road, Bristol. |
| Taylor, Thomas Henry ... ... ... ... ... | Thornbury, Gloucestershire. |
| Terry, George ... ... ... ... ... ... ... | Mells. |
| Thomas, A. Garrod, M.D., C.M. Ed. ... ... | 28 Bridge Street, Newport, Mon. |
| Thomas, G. Danford, M.D. Brussels ... ... | Park Lodge, Paddington, London, W. |
| Thomas, Jabez ... ... ... ... ... ... ... | Ty Cerrig, Swansea. |
| Thomas, R. W. ... ... ... ... ... ... ... | Saltford, Somersetshire. |
| *Thompson, George, M.D. Dur. ... ... ... | Asylum, Stapleton, Gloucestershire. |
| Thompson, John, M.D., St. And. ... ... ... | Lynton House, Bideford. |
| Thomson, G. S., M.D. Ed. ... ... ... ... | 8 College Road, Clifton. |
| Thomson, Spencer, M.D. St. And. ... ... | Ashton, Torquay. |
| *Thurston, Hugh C. ... ... ... ... ... ... | 6 Whitehall Yard, London, S.W. |
| *Tivy, W. J. ... ... ... ... ... ... ... ... | 8 Lansdown Place, Clifton. |
| Todd, W. J. ... ... ... ... ... ... ... ... | North Petherton. |
| Tomkins, Harding H.... ... ... ... ... ... | County Asylum, Gloucester. |
| Torbock, W. H. ... ... ... ... ... ... ... | Polruan, Cornwall. |
| Tosswill, Louis H., M.B., B.A. Cantab. ... | 34 West Southernhay, Exeter. |
| Trask, J. E. ... ... ... ... ... ... ... ... | Royal United Hospital, Bath. |

| | |
|---|---|
| TROTTER, LESLIE B., M.D., C.M. Ed. | Coleford, Gloucestershire. |
| TWINING, A. H. | The Knoll, Salcombe. |
| TYLEY, R. P., M.D. St. And. | Wedmore. |
| URWICK, W. | New Quay, Cornwall. |
| VACHELL, C. T., M.D. Lond. | 38 Charles Street, Cardiff. |
| VACHELL, HERBERT R., M.D., C.M. Aberd. | 15 Newport Road, Cardiff. |
| VERNON, EDWARD | Kingston, Yeovil. |
| WADE, A. LAW, M.D. Dub. | County Asylum, Wells, Somersetshire. |
| *WALDO, HENRY, M.D., C.M. Aberd. | 19 Pembroke Road, Clifton. |
| WALLACE, REV. C. HILL, M.A. Oxon. | 3 Harley Place, Clifton. |
| WALSH, DAVID H. | Ivywell House, Stoke Bishop. |
| WALTER, WILLIAM HENRY, M.D. Brussels | Knap Villa, South Petherton. |
| WARD, J. L. W. | Victoria Street, Merthyr Tydvil. |
| *WARNER, E. H., M.D., C.M. Ed. | Barton Hill House, Bristol. |
| *WATHEN, J. HANCOCKE | 16 York Place, Clifton. |
| WATSON, J. ADAM | Chudleigh. |
| WATTERS, GEORGE T. B., M.D., C.M. Ed. | Stonehouse, Gloucestershire. |
| WATTS, THOMAS | Frampton-on-Severn. |
| WAUGH, ALEXANDER | Midsomer Norton. |
| *WEATHERLY, LIONEL A., M.D., C.M. Aberd. | Bailbrook House, Bath. |
| WEBB, W. H. | Kingsbridge. |
| *WEBSTER, THOMAS | Redland Hill, Bristol. |
| *WELSH, F. F. | Rockleaze Point, Stoke Bishop. |
| WHITELEY, GEORGE W. | Downton, Wiltshire. |
| WHITFELD, W. CLARKE | 5 St. Ethelbert Street, Hereford. |
| WHYTE, ANDREW, M.B., C.M. Aberd. | Honddû House, Brecon. |
| WICKHAM, W. | Hill House, Tetbury. |
| WICKSTEED, FRANCIS W. S. | The Villa Rosa, Weston-super-Mare. |
| WIGMORE, J., M.D. Qu. Univ. I. | Twerton-on-Avon. |
| WILLCOX, R. LEWIS | Warminster. |
| WILLETT, GEORGE G. D. | 22 King Square, Bristol. |
| WILLIAMS, CHARLES | Port Isaac. |
| WILLIAMS, D. J. | Greenfield House, Llanelly. |
| WILLIAMS, H. E. | The Mount, Newport, Mon. |
| *WILLIAMS, P. WATSON, M.B. Lond. | Royal Infirmary, Bristol. |
| WILLIAMS, PETER | Ferryside, Carmarthenshire. |
| WILLIAMS, WILLIAM HENRY, M.D. Lond. | Sherborne. |
| WILTON, JOHN P. | 10 College Green, Gloucester. |
| WINTERBOTHAM, L. | Bays Hill, Cheltenham. |
| WISE, N. V. | Lindisfarne, Trowbridge. |
| WOOD, E. STANLEY | Clarence House, Pontypool. |
| WOODD, H. T. | Calstock. |
| YELF, LEONARD K., M.D. St. And. | Moreton-in-Marsh. |

### Bristol Medico-Chirurgical Society.

**President.**

F. POOLE LANSDOWN.

**President-Elect.**

L. M. GRIFFITHS.

**Honorary Secretary.**

R. SHINGLETON SMITH, M.D., B.Sc. Lond.

**Committee.**

*Past Presidents.*
R. W. COE.
AUGUSTIN PRICHARD, M.D. Berlin.
J. G. SWAYNE, M.D. Lond.
E. LONG FOX, M.D. Oxon.
JAMES G. DAVEY, M.D. St. And.
W. JOHNSTONE FYFFE, M.D. Dub.
G. F. BURDER, M.D. Aberd.
W. H. SPENCER, M.D., M.A. Cantab.

F. RICHARDSON CROSS, M.B. Lond.
NELSON C. DOBSON.
A. J. HARRISON, M.B. Lond.
W. H. HARSANT.
E. MARKHAM SKERRITT, M.D., B.S., B.A. Lond.
J. GREIG SMITH, M.B., C.M., M.A. Aberd., F.R.S.E.

Medical Men wishing to join the Society are requested to communicate with the Honorary Secretary, Dr. R. SHINGLETON SMITH, 9 Richmond Hill, Clifton. The Annual Subscription is 10/6.

*Extracts from Journal By-laws.*

4.—The *Journal* shall be delivered free to Subscribers and also to Members of the Society whose subscriptions are not in arrear.

6.—The Annual Subscription to the *Journal* for those who pay before the 1st of July shall be 5/-, post free, or 6/- if paid after that date.

Communications intended for insertion in the *Journal* should be sent to the Editor, J. GREIG SMITH, M.A., F.R.S.E., 16 Victoria Square, Clifton.

Books for Review, Exchange Journals, and communications referring to the delivery of the *Journal* should be sent to the Assistant-Editor, L. M. GRIFFITHS, 9 Gordon Road, Clifton, to whom Subscriptions are to be paid *in advance.*

Authors requiring Reprints of their Papers should communicate with the Publisher.

Cases for Binding Vols. I., II., III., and IV. are now ready, and may be obtained, price 7*d.* each (postage 2*d.* extra), upon application to J. W. ARROWSMITH, Quay Street, Bristol; or the *Journal* will be bound, including Case, for 1/3 per volume.

*Cases for binding, price 7d. each, may be had of the Publisher,*
J. W. ARROWSMITH, *Quay Street, Bristol.*

### Exchange-List.

### AFRICA.

### CAPE COLONY.

*Weekly.*

*The South African Medical Journal.* Cathcart: W. Darley-Hartley.

### AMERICA.

### ARGENTINE REPUBLIC.

*Monthly.*

*Anales del Círculo Médico Argentino.* Buenos Ayres: M. Biedma.

### CANADA.

*Monthly.*

*Canada Medical and Surgical Journal.* Montreal: Gazette Printing Company.

### PERU.

*Monthly.*

*La Crónica Médica.* Lima: Carlos Prince.

### UNITED STATES.

*Weekly.*

*The Cincinnati Lancet-Clinic.* Cincinnati: 281 West 7th Street.

*The Journal of the American Medical Association.* Chicago: 65 Randolph Street.

*The Medical and Surgical Reporter.* Philadelphia: 115 South Seventh Street.

*The Medical Record.* New York: William Wood & Co.

*The New York Medical Journal.* New York: D. Appleton & Co.

*Fortnightly.*

*Philadelphia Medical Times.* Philadelphia: J. B. Lippincott & Co.

*The American Practitioner & News.* Louisville: John P. Morton and Company.

*Twice a month.*

*The Medical Age.* Detroit: George S. Davis.

*Monthly.*

*Abstract and New Books.* Weston: H. H. Howe, M.D.

*Index Medicus.* Detroit: George S. Davis.

*Journal of Cutaneous and Genito-Urinary Diseases.* New York: William Wood & Company.

*New Orleans Medical and Surgical Journal.* New Orleans: 33 Carondelet Street.

*Pacific Medical and Surgical Journal and Western Lancet.* San Francisco: W. S. Duncombe.

*The American Journal of Ophthalmology.* St. Louis: J. H. Chambers & Co.

*The Medical Analectic.* New York: G. P. Putnam's Sons.

*The Obstetric Gazette.* Cincinnati: 281 West 7th Street.

*The Polyclinic.* Philadelphia: P. Blakiston, Son & Co.

*Monthly.*

*The Saint Louis Medical and Surgical Journal.* Saint Louis: 2622 Washington Avenue.

*The Therapeutic Gazette.* Detroit: George S. Davis.

*Quarterly.*

*The American Journal of the Medical Sciences.* Philadelphia: Lea Brothers & Co.

*Yearly*

*Annual of the Universal Medical Sciences.* Philadelphia: 1632 Chestnut Street.

*Transactions of the American Surgical Association.* Philadelphia: P. Blakiston, Son & Co.

*Occasionally.*

*Transactions of the New York Academy of Medicine.* New York: 12 West 31st Street.

### ASIA.

### INDIA.

*Monthly.*

*The Indian Medical Journal.* Calcutta: W. Newman & Co., Ld.

### JAPAN.

*Monthly.*

*The Sei-I-Kwai Medical Journal.* Tokio: Z. P. Maruya & Co.

### AUSTRALASIA.

### AUSTRALIA.

*Monthly.*

*The Australasian Medical Gazette.* Sydney: L. Bruck.

*The Australian Medical Journal.* Melbourne: Stillwell & Co.

### EUROPE.

### BELGIUM.

*Monthly.*

*Bulletin de l'Académie royale de médecine de Belgique.* Brussels: F. Hayez.

### BRITISH ISLES.

*Weekly.*

*British Medical Journal.* London: 161 A Strand.

*The Hospital.* London: Whiting & Co.

*The Lancet.* London: 423 Strand.

*Monthly.*

*Annals of Surgery.* London: Baillière, Tindall & Cox.

*The Birmingham Medical Review.* Birmingham: Cornish Bros.

*The Journal of Laryngology and Rhinology.* London: J. & A. Churchill.

*The London Medical Record.* London: Smith, Elder, & Co.

*The Medical Chronicle.* Manchester: John Heywood.

*The Practitioner.* London: Macmillan & Co.

*Monthly.*

*Edinburgh Medical Journal.* Edinburgh: Oliver and Boyd.

*The Glasgow Medical Journal.* Glasgow: Alex. Macdougall.

*The Dublin Journal of Medical Science.* Dublin: Fannin & Co.

*Quarterly.*

*The Asclepiad.* London: Longmans, Green, & Co.

*The British Gynæcological Journal.* London: Smith, Elder, & Co.

*Half-yearly.*

*The Liverpool Medico-Chirurgical Journal.* Liverpool: Medical Institution.

*The Retrospect of Medicine.* London: Simpkin, Marshall, & Co.

*Yearly.*

*The Medical Annual.* Bristol: John Wright & Co.

*The Year-Book of Treatment.* London: Cassell & Company, Limited.

*Year-Book of Scientific and Learned Societies.* London: C. Griffin & Co.

*Transactions of the Academy of Medicine in Ireland.* Dublin: Fannin and Company.

DENMARK.

*Weekly.*

*Hospitals-Tidende.* Copenhagen: Jacob Lund.

FRANCE.

*Three times a week.*

*Gazette des hôpitaux.* Paris: 4 Rue de l'Odéon.

*L'Union médicale.* Paris: 11 Rue de la Grange-Batelière.

*Weekly.*

*Bulletin de l'Académie de médecine.* Paris: G. Masson.

*Gazette médicale de Paris.* Paris: 8 Place de l'Odéon.

*Le Progrès médical.* Paris: 14 Rue des Carmes.

*Twice a month.*

*Gazette de Gynécologie.* Paris: O. Doin.

*Monthly.*

*Nouvelles Archives d'Obstétrique et de Gynécologie.* Paris: Félix Alcan.

*Revue mensuelle de Laryngologie, d'Otologie et de Rhinologie.* Paris: 8 Place de l'Odéon.

GERMANY.

*Twice a week.*

*Deutsche Medizinal-Zeitung.* Berlin: Eugen Grosser.

*Weekly.*

*Medicinische Bibliographie.* Leipzig: Breitkopf & Härtel.

GREECE.

*Weekly.*

Γαληνὸς. Athens: N. G. Passare.

HOLLAND.

*Weekly.*

*Nederlandsch Tijdschrift voor Geneeskunde.* Amsterdam: F. van Rossen.

ITALY.

*Monthly.*

*Lo Sperimentale.* Florence: 35 Via degli Alfani.

NORWAY.

*Monthly.*

*Norsk Magazin for Lægevidenskaben.* Christiania: Th. Steens.

PORTUGAL.

*Weekly.*

*A Medicina Contemporanea.* Lisbon: José Antonio Rodrigues & Ca

ROUMANIA.

*Monthly.*

*Spitalul.* Bucharest: Stefan Mihalescu.

RUSSIA.

*Weekly.*

*Vrach.* St. Petersburg: C. Ricker.

SPAIN.

*Weekly.*

*El Siglo Médico.* Madrid: Magdalena, 36, 2°

SWEDEN.

*Quarterly.*

*Nordiskt Medicinskt Arkiv.* Stockholm: P. A. Norstedt & Söner.

SWITZERLAND.

*Monthly.*

*Illustrirte Monatsschrift der ärztlichen Polytechnik und Centralblatt der orthopädischen Chirurgie.* Berne: Schmid, Francke & Co

### *BOOKS AND PAMPHLETS RECEIVED.*

From David Torres Aguirre, Lima:
*El Monitor Médico.* Año Segundo. Num. 13, 14.

From Bigelow Brothers, Buffalo:
*The Medical Press of Western New York.* Vol. II. Nos. 1—3. 1887.

From D. O. Haynes & Company, Detroit:
*The Pharmaceutical Era.* Vol I. No. 1. 1887.

From 93 Fulton Street, New York:
*New York Medical Abstract.* Vol. VI. No. 11. 1886.

From J. B. Baillière et Fils, Paris:

*Bibliographie des Sciences médicales.* 1887.

From 16 Rue Clément Marot, Paris:

*Revue générale de clinique et de thérapeutique.* No. 1. 1887.

From Th. Steens, Christiania:
*Dilatatio Ventriculi.* Unger Vetlesen. 1887.

From 4 Rua do Carmo, Oporto:

*Revista de Medicina e Cirurgia.* No. 1. 1887.

From Dr. W. Jaworski, 19 Rue St. Jean, Cracow:
*Ueber Wirkung, therapeutischen Werth und Gebrauch des neuen Karlsbader Quellsalzes, nebst dessen Beziehung zum Karlsbader Thermalwasser.* Von Dr. W. Jaworski. Separatabdruck aus Dr. Wittelshöfer's *Wiener Mediz, Wochenschrift.* 1886.
*Beitrag zur klinischen Mikroskopie des Mageninhaltes.* Von Doc. W. Jaworski. Separat-Abdruck aus dem *Centralblatt für klinische Medicin.* 1886.

From Carl Ricker, St. Petersburg:

*St. Petersburger Medicinische Wochenschrift.* No. 1. 1887.

From J. Sol Torrens, Lérida:

*Boletin Clinico.* Año I. Núm. 5—7.

From J. & A. Churchill, London:
*Alpine Winter in its Medical Aspects.* By A. Tucker Wise, M.D. Third Edition. 1886.
*Causation and Treatment of Congenital Club-foot.* By Frederick Churchill, M.B. 1887.
*On Aphasia.* By James Ross, M.D., LL.D. 1887.
*Litholapaxy in Male Children and Male Adults.* By Surgeon-Major D. F. Keegan, M.D. 1887.
*Leçons sur les Auto-Intoxications dans les Maladies.* Par Ch. Bouchard. (Paris: F. Savy.) 1887.
*Vegetable Biology.* By Thomas W. Shore, M.D., B.Sc. 1887.
*Aural Surgery.* By Sir William R. Dalby, M.B. 1887.
*Pulmonary Consumption.* By James Weaver, M.D. 1887.
*Medical Galvanism.* By Herbert Tibbits, M.D. 1887.
*Oxygen as a Remedial Agent.* By Charles J. Smith. 1887.

From H. K. Lewis, London:
*On Fevers.* By Alexander Collie, M.D. 1887.
*Gout.* By Robson Roose, M.D. Third Edition. 1887.
*Physical Diagnosis of Lung Diseases.* By J. Magee Finny, M.D. 1887.
*A Text-Book of Medicine.* By Dr. Adolph Strümpell. 1887.

From Young J. Pentland, Edinburgh:
*Atlas of Venereal Diseases.* By P. H. Maclaren, M.D. Fasc. X. 1886.

### *Abdominal Surgery*

BY

J. GREIG SMITH, M.A., F.R.S.E.,

*Surgeon to the Bristol Royal Infirmary; late Examiner in Surgery Aberdeen University.*

---

**Extra Demy 8vo. Pp. 606. Price 15s.**

*London:* J. & A. CHURCHILL. *Bristol:* J. W. ARROWSMITH.

---

### OPINIONS OF THE PRESS.

*British Medical Journal.*—"Heartily congratulating the author . . . we hope we may see several editions."

*The Lancet.*—"We consider the book a success, and anticipate for it a favourable reception."

*British Gynæcological Journal.*—"Dr. GREIG SMITH is to be heartily congratulated on his valuable work, which is sure to pass through many editions, and which reflects the greatest credit on himself and provincial surgery."

*Dublin Journal of Medical Science.*—"We think this book not only highly creditable to its author, but to English Surgery. No one will read it without feeling that he has before him the personal work and experience of a surgeon of great ability and accomplishments."

*The Medical Chronicle.*—"All must read it with interest, and instruction, and pleasure; for it is fresh, and new, and readable. To the average reader of medical journals it will be almost the discovery of a new world, and few, if any, can be in a position not to find in it the suggestiveness which stimulates thought, and leads to the evolution of new experience."

*New York Medical Journal.*—"Its author has been singularly happy in his method and manner of performing his task. The book is a . . . model of conciseness without the sacrifice of necessary detail, and clearness of description without verbosity."

